# Round the Hospitals

**Published:** 1919-05-10

**Authors:** 


### ROUND THE HOSPITALS.

On Thursday, May 1, Viscount Goschen introduced into the House of Lords a Bill to provide for the training and registration of nurses, which represents and is supported by the opinion of those to whom the nation owes most of its trained nurses as they exist to-day. The Bill has been read a first time, and has been printed and circulated.

The results of the nominations for the twelve vacancies on the College of Nursing Council indicate that members must bestir themselves if they wish to see nurses, as opposed to matrons, representing them on the Council in the ensuing year. The total number of candidates nominated is 108, of whom four are nurses, five sisters, one health visitor, thirteen doctors, two barristers, and the remainder, eighty-three, are matrons. Whoever else may be elected, members of the College should see to it that four of the vacancies are filled by the four nurses who have been nominated. The voting papers will

**ROUND THE HOSPITALS**—*(continued)*.

shortly be issued, and it will then be in the hands of the nurse-members to decide by their votes whether the composition of the College Council shall reflect the alleged desire for government of the nurses, by the nurses, for the nurses, or whether they prefer to be represented wholly and exclusively by the matrons and doctors whom they have nominated in such an overwhelming majority. A letter from one of the nurse-candidates will be found on page viii, with the notice of the Edinburgh meeting.

At the open-air Investiture held at Buckingham Palace on May 3, the King personally conferred the decoration of the Royal Red Cross, Second Class, on Sister Marie Fielding, Sister Dora Lovell, and Sister Eva MacDonald, of the Australian A.N.S. These ladies were subsequently received by Queen Alexandra at Marlborough House.

The plan of arrangements for the funeral of Miss Edith Cavell has been issued by the Executive Committee. The body will arrive at Dover on the 14th inst., and the programme on the following day will be as follows: 7.30 A.M. train leaves Dover for Victoria; 11.0 A.M. train reaches Victoria; 11.45 A.M. funeral procession arrives at Westminster Abbey; 12.45 P.M. procession leaves the Abbey; 2.30 P.M. special train leaves Liverpool Street for Norwich; 4.50 P.M. arrival at Norwich; 5.0 P.M. procession leaves for Norwich Cathedral; 5.30 P.M. services at the Cathedral. After leaving Westminster Abbey the route to Liverpool Street will be by way of: Parliament Square, Bridge Street, Victoria Embankment, Blackfriars, Queen Victoria Street, Mansion House, Broad Street, Liverpool Street. The constitution of the funeral *cortège* will be entirely military, the War Office being in charge both in London and at Norwich.

There is to be a monument to Miss Edith Cavell in the Ecole des Infirmières, Brussels, where she initiated and carried out as matron the training of Belgian nurses. The governing body of the institution has voted the sum of £1,000 for a memorial statue, designed by M. Paul Dubois, to be placed in the garden in front of the school.

The Nurses' Bazaar at Devonshire House on May 1, 2, and 3, brought together a unique gathering. It was opened by Queen Alexandra, who was accompanied by Princess Victoria and a distinguished company. The great central marquee was admirably decorated and arranged. Queen Alexandra's stall, under Lady Arbuthnot Lane, on the right of the entrance, was resplendent with Japanese and Chinese embroideries, and showed also many very fine curios, among which a quaint nightdress of Queen Victoria amused and interested the throng as much as anything. St. Thomas's stall, charmingly arranged under a Norman arch, with its symbolic hangings, began business with £600 in hand as the result of an appeal to those connected with the Hospital, besides £55 15s. 6d. collected by "Old Nightingales." The University College stall, a symphony in white and blue, had some very beautiful and delicate needlework, all by the nurses and much admired by Queen Alexandra, who purchased here some embroidered crepe de chine underclothes. The Royal Free Hospital, besides running with admirable business ability the tea hut, had a dainty display of baby clothes, and baskets exquisitely made and embroidered by the nurses. The East-End hospitals, twelve in number, had some interesting paintings contributed by the Duchess of Marlborough and much attractive needlework. The Middlesex Hospital stall also made a speciality of paintings and was very well stocked besides in every particular. It was noticeable that bazaar "rubbish" was conspicuously absent from every stall.

Perhaps no stall had enjoyed better preliminary organisation than that of the Hampstead hospitals. The amount contributed before the bazaar began totalled over £900, and the fund will be kept open all the summer under the treasurership of Miss Olga Nethersole, Heathland's Lodge, Hampstead, where cheques will be welcomed. Some fine replicas of an Italian Tazza, the original of which is in the South Kensington Museum, were presented to this stall by Miss Jacobs in memory of her brother, and one of these was bought by Queen Alexandra. The Chelsea group of hospitals showed some lovely Wedgwood china presented by the firm and speedily sold out, as well as some capital costume dolls. Other hospitals represented were King's College, Guy's, the Weir Hospital, Balham, the Freemason's Hospital (which collected over £90 beforehand), St. Gabriel's, Camberwell, St. Bartholomew's, and the West London. The No. 12 British Red Cross working parties had an enticing display of useful things, including a pile of men's pyjamas at 10s. 6d. a pair which were a real bargain. Lady Cowdray did good business at a well-furnished provision stall, and there were many other attractions too numerous to mention. Altogether it was an exceptionally well-managed and well-provided sale of many-sided and genuine interest and value. We understand the proceeds more than totalled the £5,000 its promoters set out to gain.

The services held at St. Martin's-in-the-Field on May 1, 2, and 3 were a refreshing interlude in the business of buying, selling, sight-seeing, and other fatigues provided for nurses during the week. The Rev. A. Lombardini took for his subject on all three days the Divine Legacy left by our Lord to his executors, among whom nurses were to be especially reckoned. The address on May 2 may be mentioned as particularly helpful and suggestive, the legacy of joy which Christ left to His followers being brought before a deeply-interested group of nurses. The preacher laid stress on the spirit of joy and happiness as one of the great secrets of nurses through which, often unconsciously, they bring harmony to their patients, and he spoke with conviction of the life of Jesus Christ as "undoubtedly the happiest life which has ever been lived on earth." Mr. Lombardini looks forward to

**ROUND THE HOSPITALS—(*continued*).**

securing a nurses' church in Central London, where a memorial may be erected to the women who have given their lives to the profession.

One of the most interesting conferences held at the Mortimer Hall last week dealt with the subject of pensions for nurses. Lady Bathhurst, advocating the establishment of one comprehensive Fund for aiding nurses in distress and granting their pensions, outlined a scheme through which the Nation's Fund for Nurses would form a benefit society, to which nurses would contribute instead of to the National Insurance Fund. She contended that all registered nurses ought to be secured disablement allowance as well as pensions. Mr. Louisdick, who said that the present rate of pay of many nurses was a disgrace, gave it as his opinion that the person who was not thrifty on a small income would never be thrifty on a larger one. The main thing was to cultivate the habit of thrift early in the career.

Immense gratification has been caused by the announcement that the Territorial Force is to continue under a new constitution, and that the Territorial Nursing Service, which worked so well during the war, is to be maintained. It was the T.F.N.S., working under officers already well known, capable of indefinite expansion and ready at a moment's notice, which saved the situation when war broke out, and the need for a thousand-fold increase in the number of Army nurses became pressingly, overwhelmingly evident. The nurses of the T.F.N.S. feel with great force what Sir Douglas Haig said of the Territorial Force generally, the desire to keep up the spirit of comradeship which the war brought with it. And they, too, realise very clearly the truth of his words: "Those who served the country we can never recompense with anything commensurate to what they have given to the country." Still, we might try.

We are far from convinced that the National Council of Women received an authoritative mandate from the nursing profession to inquire into "the economic position of nurses," but even if we were, we should demur to the inquisitorial *questionnaire* issued by the Special Committee which has taken this matter in hand, to the chief London and provincial hospitals, cottage hospitals, infirmaries, and hospitals controlled by the Metropolitan Asylums Board. They comprise eighty-one questions, ranging in importance from full particulars of all courses of lectures, to the provision of sufficient footstools in the nurses' sitting-rooms. The questions demand information about the most minute details of the domestic organisation, and are more suited to form the private notes of a Government inspector of workhouses than to procure reliable data on the position of nurses in voluntary and municipal institutions. If anything were needed to expose the evils of allowing outsiders to intervene in professional matters, this *questionnaire* would prove convincing. Is this the kind of harrying to which the representatives of the Seven Societies are looking forward under a Registration Act? We note that the representatives of the College of Nursing have withdrawn from the Special Committee appointed at the Conference held by the National Council of Women. The matter of nurses' pay is under consideration by a Special Committee appointed by the nurses themselves.

Opinion is divided in Scotland on the question of whether women as women ought to be entitled to constitute one of the consultative councils formed in connection with the Health Ministry. Six women's associations are in favour of a special women's council and five are against it. In this country the appointment of a special women's council has been vetoed by Dr. Addison. There is much to be said for the contention that "the ripest experience, the best brains, and the highest skill" should be represented on these councils, quite irrespective of sex, and we trust the event may prove that the claims of women to possess some of these excellencies may be vindicated. Queen Victoria's Jubilee Institute for Nurses in Scotland has petitioned Mr. Robert Munro, Secretary for Scotland, through an influential deputation, for the inclusion of a fully-trained nurse as a member of the Scottish Board of Health, and has received a non-committal reply.

A graceful message was sent to Princess Mary by the members of the Whitefriars Club on the occasion of their ladies dinner at the Trocadero Restaurant on May 3, Sir Robert Hudson, expressing the appreciation felt by all present for the work performed by women in the war, and more particularly by members of the Nursing Service, offered deep and respectful admiration to Her Royal Highness for the example she had set as a V.A.D. member. The Princess, in telegraphing her thanks to the club, said: "I share their admiration of the work which has been done by the nursing services during the war, and I am very proud of belonging to the V.A.D.s."

Great changes were inaugurated in the nursing department at St. George's Hospital on May 1. Increases of salary were made, commencing at an annual cost of £715 and estimated to cost £1,470 a year in the course of time. The working hours of the nursing staff were reduced and arrangements set on foot to reduce them still further when the needful addition to numbers can be made. The salaries as raised stand as follows:—Assistant matron, £100-£150; sister housekeeper and Nurses' Home sister, £70-£100; night superintendent, £70-£90; out-patient sister, £75-£100; sister tutor, £70-£90; sister of the Electro-Therapeutic department, £100-£150; ward sisters, £50-£80; staff nurses, £35; probationers, £15, £20, £25, with uniform and laundry. We feel confident that the public will contribute the means to meet this extra expenditure which is now recognised by all to constitute an act of justice rather than of generosity.

**ROUND THE HOSPITALS—(*continued*).**

Many of the North Country hospitals are reorganising their nursing departments, and among them the Bradford Royal Infirmary, long noted for its efficient administration, has inaugurated a specially good scheme of alterations in pay, off-duty time, and system. The latter is, no doubt, the most important in the real interests of probationers and we learn with pleasure that preliminary training is shortly to be given to candidates and that the teaching is to be placed under the direction of a sister tutor, who will also be home sister. Each member of the staff is to have one day a week off-duty, and the working hours will be reduced to sixty a week. Sisters are to start at a salary of £45, and rise by yearly increments of £5 to £65. An advanced course has been arranged for staff nurses who remain for a fourth year at a salary of £40. It comprises instruction in massage, electricity, *x*-ray work, the treatment of venereal disease, etc., and in housekeeping and administrative departments.

---

At the nineteenth annual meeting of the Nelson Hospital at Wimbledon, the chairman announced that "their most admirable and overworked matron" had been relieved to some extent by the appointment of an assistant housekeeper, and that "the hours of the nursing staff, which used to be more than they ought," had been reduced to eight a day. He rightly declared that the work of a nurse was extremely strenuous, and that if she did eight hours she did well. An electrical department is being fitted up, and in view of the approaching enlargement of the institution the Council have acquired cottages in the neighbourhood. The only unsatisfactory feature in the report is the meagreness of the annual contributions. This surely points to some defect in advertising the needs of the hospital. Dr. Loudon Strain referred to the "sweated labour" of trained nurses, and expressed his satisfaction that nurses in future would not be worked as they had been.

---

Among the letters of Florence Nightingale sold at Sotheby's recently are some written from Scutari during the Crimean War. She mentions that out of 108 nurses sent out from England, twelve were dismissed on account of intoxication and fifty-two for other causes. Her remarks on the situation might possibly be deemed too tolerant by a modern matron: "Intoxication, tacitly admitted unavoidable among nurses in London hospitals, must in military hospitals be sternly checked."

---

The nurses' hours on duty, which now total sixty-five per week, at the Royal Portsmouth and Gosport Hospital are to be reduced to fifty-five, an average of some eight hours a day, in accordance with a scheme worked out by the Matron. The reform demands an increase of the staff from thirty-four to forty-one, or seven additional nurses. The alteration is intensely significant in view of the reason alleged for it. The Management Committee are unanimously of opinion that unless this reform is carried out it will be impossible to obtain probationers or retain nurses in the establishment. The rapidity with which these concessions are spreading throughout the voluntary institutions of the country testifies to a universal awakening to the stern facts of the nurse's life, not only among sympathisers and reformers, but also among the great body of young women on whom the nursing profession depends for its continued existence.

---

The Scottish branch of the British Red Cross Society decided, as we announced a short time back, to form their own scheme for affording additional training to their workers as required on demobilisation. This scheme differs principally from that formulated by the British Red Cross Society in England and Wales in that it admits the claims of all those who have served in Naval, Military, and Red Cross Hospitals during the War, that is, of trained nurses as well as of members of Voluntary Aid detachments. Applications from other Red Cross workers and the claims of the Q.A.I.M.N.S. and Reserve, the T.F.N.S., and the nurses who have worked in civil hospitals which treated sailors and soldiers during the War "will be considered on their merits." Among the kinds of training for which grants in aid will be available are some which appeal strongly to trained nurses, such as midwifery, health visiting, massage, electricity, and Swedish remedial exercises, pharmacy, and domestic science in connection with hospital work. Nurses who have worked under the Scottish branch and desire to enlarge their boundaries of knowledge should write for a form of application to the Secretary, B.R.C.S., 86 St. Vincent Street, Glasgow, not later than June 15, 1919.

---

Among plans for war memorials one, particularly worthy of note as an example to be followed elsewhere, is that adopted by St. Aidan's Church, Stratford. The congregation has decided to raise the necessary funds to provide a trained nurse for the district who will be available for any cases of need, whether belonging to the Church or not. This live memorial is one which the fallen themselves might have chosen.

---

Hospital workers will find much to interest them in the new film play called "Women Who Win," in which the Queen and Queen Alexandra are both represented. Their Majesties' assent was graciously given to the petition that they would permit a film to be taken of scenes in which they crown the successful worker's career. Queen Mary is depicted during an inspection of an aeroplane factory at the moment when the heroine is presented to her. Queen Alexandra appears in the conservatory of Marlborough House among sisters who are recipients of the Royal Red Cross.

